#  Randomized Trial of the Effect of Magnesium Sulfate Continuous Infusion on IL-6 and CRP Serum Levels Following Abdominal Aortic Aneurysm Surgery

**Published:** 2016

**Authors:** Mojtaba Mojtahedzadeh, Legese chelkeba, Mona Ranjvar- Shahrivar, Atabak Najafi, Majid Moini, Farhad Najmeddin, Kourosh Sadeghi, Khosro Barkhordari, Azin Gheymati, Arezoo Ahmadi

**Affiliations:** a *Department of Anesthesiology and Critical Care, Sina hospital, Faculty of Medicine, Tehran University of Medical Sciences, Tehran, Iran. *; b *Department of Clinical Pharmacy and Pharmaceutical Science Research Center, Tehran University of Medical Sciences, Tehran, Iran.*; c *Faculty of pharmacy and Pharmaceutical Research Center, Tehran University of Medical Sciences, Tehran, Iran. *; d *Department of Surgery, Sina hospital, Faculty of Medicine, Tehran University of Medical Sciences, Tehran, Iran.*; e *Department of Anesthesiology and Critical Care, Tehran Heart Center, Faculty of Medicine, Tehran University*

**Keywords:** Abdominal aortic aneurysm (AAA), Magnesium Sulfate (MgSo_4_), Interleukin 6 (IL-6), C- reactive protein (CRP)

## Abstract

Abdominal aortic aneurysm (AAA) is widely considered as the disease of elderly white men. Inflammation is one of the most well-known mechanisms involved in the pathogenesis of AAA. Magnesium is one of the most important minerals in the body with established anti-inflammatory effects. In this study, we aimed to investigate the impact of Mg loading following AAA surgery on two inflammation markers, IL-6 and CRP, as well as patientʼs outcome.

This study was conducted as a randomized clinical trial on 18 patients (divided into two groups) after surgical correction of Acute Aortic Aneurysm (AAA). All the patients admitted in ICU ward of Sina Hospital. In intervention group, 10 g of MgSO_4_ has been infused through 12 h. The control group has not received the intervention. IL-6 and CRP were measured and compared at times 0, 12, 24 and 36 h. The patients were monitored for 36 h.

After intervention, the differences of heart rate and APACHE II score were not statistically significant between intervention and control groups (P = 0.097 and P = 0.472, respectively). IL-6 levels decreased consistently in both groups after inclusion in the study. However, IL-6 level was significantly less in intervention group early after the end of MgSO_4_ infusion comparing with control group (P = 0.01). Likewise, the CRP level decreased significantly after inclusion in the study (P = 0.005). However, these changes were not significant between intervention and control groups (P = 0.297).

According to the results of this study, continuous infusion of MgSO4 after AAA surgery may provide IL-6 suppression.

## Introduction

Abdominal aortic aneurysm (AAA) is relatively common in elderly men and is responsible for 1.3% of death among men aged 65–85 years old in developed countries (1, 2). Open surgery is the conventional choice in the case of aneurysm rupture. This surgery requires aorta to be clamped, which can increase the risk of splanchnic ischemia. Splanchnic ischemia is a well-known complication of aortic surgery (3) and triggers the release of pro-inflammatory cytokines such as IL-6 (3). There is growing number of evidence that suggests aortic aneurysms secrete IL-6 into the circulation (4). High levels of IL-6 in blood stream provoke severe systemic adverse reactions in different organs especially lung, kidney and myocardium, leading to multiple organ dysfunction syndrome (MODS) (3). Moreover, association of prolonged or enhanced levels of IL-6 and poor prognosis in postoperative period has been expressed in the recent studies. Hence, decreasing IL-6 level may be followed by better outcome, as it has been shown that pretreatment with antibodies against IL-6 prevents remote organ injury in experimental models (3).

Magnesium (Mg) is the second-most common cation in cellular systems. It regulates hundreds of enzymes which are involved in different cellular process and its progressive deficiency is strongly associated with increased mortality (5). Mg deficiency is more frequent among ICU patients (6). There is growing evidence that Mg has important function in regulating immune response and Mg deficiency may play a role in inflammation (7). Recent studies suggest that increased serum ionized Mg^2+^ may inhibit IL-6 production after reperfusion and may prevents its subsequent adverse effects (5).

On the basis of these data, we sought to determine the relationship between IL-6 and administration of magnesium. It is hypothesized that continuous infusion of magnesium would be effective and safe for reducing IL-6 secretion in the blood stream. As it has been proved that IL-6 has an important role in regulation of CRP, the combined use of interleukin-6 and CRP levels suggested as indicators of inflammation with better predictive value (8).

## Subjects and Methods

*Study Area and Period*

Patients admitted after vascular surgery to the general ICU ward of Sina Hospital affiliated to Tehran University of Medical Sciences (TUMS),Tehran, Iran from September 2011 to May 2012 were screened for study eligibility and randomly assigned in either intervention or control group. 

*Study design and randomization*

This study was a randomized open labeled clinical trial that was approved by TUMS review board and conducted on patients admitted to general ICU of Sina Hospital of Tehran University of Medical Sciences as a result of abdominal aortic aneurysm and underwent surgical repairing to determine the effect of magnesium sulfate on IL-6 and CRP. Computer-generated random numbers were utilized for randomization either to the magnesium sulfate or control. We included 18 patients randomized equally into intervention and control groups. Within this one year period we had only 18 patients that fulfill our criteria that were very tight and restricted. 

*Inclusion and exclusion criteria*

Inclusion in the study required the patient to have major abdominal aortic aneurysm repair procedures. Patients with the following conditions were excluded from the study: known renal impairment (defined as glomerular filtration rate (GFR) < 50% baseline or urine output < 0.5 mL/Kg/h within 12 h ), acute renal failure(defined as serum creatinine increase by ≥0.3 mg/dL within 48 h or urine output decrease by ≤500 mL/d or 25 mL/h for 4 h), major cardiac, pulmonary or hepatic diseases (defined as the presence of hepatic cirrhosis, hepatic encephalopathy or concentration of serum transaminases greater than three times the upper limit of normal range); a moribund condition at admission; hematologic diseases, neuromuscular diseases, patients who had taken endothelial active drugs (e.g. angiotesin-converting-enzyme inhibitors, corticosteroids and statins) (9), hypomagnesemia, hypermagnesemia, previous magnesium intake, pregnancy or lactation and age below 18 years old. Patients with hypotension (Mean Arterial Pressure (MAP) <50 mm Hg) or bradycardia (heart rate <60 beat/s) were excluded as well. 

*Data collection*

Baseline levels of blood glucose, magnesium, serum electrolytes, urea, creatinine, Blood Urea Nitrogen (BUN), hemoglobin, platelet and WBC counts, activated Partial Thromboplastin Time (aPTT), Prothrombin Time (PT), International Normalization Ratio (INR), Erythrocyte Sedimentation Rate (ESR) and albumin were obtained and electrocardiography was performed before infusion. Patients were checked for changes in blood pressure or development of arrhythmia. Each group received standard treatments including volume resuscitation, utilizing 0.9% normal saline and albumin. Demographic data, length of ICU stay, administered medications and also surgical and medical history were drawn from each patient record. The severity of illness was inspected using the Acute Physiology and Chronic Health Evaluation (APACHE II) scoring system.

*Analytical methods*

Intervention group involved patients who received 50 mL of 20% MgSO4 solution. This amount is equivalent to 10 g of MgSO4which was diluted in 500 mL of NaCl (0.9% v/v) and was infused in 12 h. Control group received no intervention during the study period. Five mL venous blood samples were taken from a central catheter with a heparinized syringe before starting infusion (Baseline sample), after 12, 24 and 36 h post administration to measure IL-6 and High-Sensitive CRP(HS-CRP). The plasma was separated after centrifugation at 4500 rpm for 15 min and stored at −80 °C until the time of analysis. IL-6 and HS-CRP levels were measured by commercially available Electro Chemiluminescent immunoassay (ECLIA) kits (Roche, Germany) utilizing Elecsys® analyzer (Roche, Germany).

*Study End Points*

Primary endpoints were the plasma level of IL-6 and CRP, whereas the secondary end points were changes in APACHE II score, lengths of ICU stay and mortality rate reduction by the drug given to the patient compared to the control groups.

*Ethical consideration*

All potential participants or their knits were consulted for consent prior to sample collection and all procedures were realized as per the guidelines for biomedical research (The study conducted according to the declaration of Helsinki regarding studying human subjects and Informed consent was obtained from either the patients we (10)code number (89-230-2717). 

The investigator, head of the medical institution, promptly submitted the protocol to the applicable ethical review boards. The patients had full right to withdraw at any time from the trial if they didn’t accept it. An identification code assigned by the investigator to each patient was used instead of patient´s name to protect patient´s identity when reporting trial related data ((10) code number (89-230-2717).

*Statistical analysis*

All the analyses were carried out by SPSS statistical package, version 20.0 for windows. Chi-square test was used to compare the proportions. Unpaired t test and Mann– Whitney test were used to assess differences between the treatment groups at each time point for parametric and nonparametric variables, respectively. To assess differences between the time points in each treatment group, repeated-measure one-way analysis of variances and nonparametric analysis of variances (Friedman test) were used to analyze changes in biomarkers level. Regarding large difference size between baseline and follow up levels of IL6, percentage change was calculated to reduce variance within intervention and control groups while performing analysis. P–values less than 0.05 were regarded as statistically significant.

## Results

Eighteen patients including 15 (83.3%) male and 3 female were randomized equally to either intervention or control group. The median age was 74 (ranging 40 to 92) years old. The patients in each group were similar at baseline point regarding their age, gender and APACHE II score (Table 1.). Median ICU length of stay were 8 ± 13.05 and 5 ± 1.7 in intervention and control groups, respectively (P = 0.258). In addition, ICU mortality rates were 22.2% in both groups.

**Table 1:** Demographic characteristics of subjects at the baseline point (0 h).

**Intervention group (n=9)** *		**control group (n=9)** *	**P value** #
Age (year)	75.89±2.83	72.56±4.50	0.540
Male (%)	100%	66.7%	0.206
APACHE II score	15.66±1.01	14.66±1.56	0.599
HR(0 h)	100.33±6.35	85.55±4.62	0.078

* Data shown as mean ± SE. Heart rate (HR),

# p value <0.05 considered as significant.

**Table 2:** IL-6 level values at different times in intervention and control groups

**IL-6 (pg/mL)**	
**Int.(mean±SE)**	**Ctrl. (mean±SE)**
1518.00±307.46	808.42±266.27
361.33±121.63	421.10±105.34
444.50±111.59	285.05±96.64
549.92±197.69	212.42±171.20

P: P-value; t: total; T: time (h); Int.: Intervention group; Ctrl.: Control group; 1: comparison of each time group relative to the previous time; P- value <0.05 considered as significant.

The mean heart rate (HR) decreased from 100.33 ± 6.35 to 98.5 ± 4.19 (p = 0.609) in the intervention group and increased from 85.55 ± 4.62 to 93.11 ± 5.10 (p = 0.256) in the control group at 24 h. However, the differences were not statistically significant when compared between these two groups (P = 0.097). Likewise, APACHE II score at 24 h were 16.12 and 14.22 in the intervention and control groups, respectively (P = 0.472).

IL-6 levels decreased consistently in the whole sample population after inclusion in the study with the lowest amount at T36 (Table 2. IL-6t = 381.17 ± 130.76 pg/mL). This decrease was significant at T12 and T24 compare to the IL-6 level at their corresponding previous step (Table 2. P = 0.001 and P = 0.007, respectively). Nevertheless, the difference of IL-6 level between intervention group and control group was significant only at T12 (P = 0.042). In addition, trend of reduction in IL6 level in intervention group is not continued through the follow up (chart 1). Adjusting to the baseline we used percentage change from baseline which was -68.61 ± 9.27 at T12 in intervention group comparing to -34.37±8.78 in control group which was statistically significant (P = 0.019). Percentage change was also used to describe changes of T24 from T12. In intervention group percentage change returned to positive value (+14.32 ± 10.13) at T24 in contrast with reduction trend continuation in control group (-23.23 ± 14.48) although not statistically significant (P = 0.058).

Likewise, CRP level decreased and the lowest amount was at T24 (Table 1. and Table 2. CRP t = 42.00 mg/L). The decrease rate at T24 was significant relative to the T12 (P = 0.004). However, the differences were not significant either between or within each of the intervention or control groups (Table 2.) and no correlation observed between CRP and IL6 levels.

Serum levels and percentage change of IL-6 and CRP serum levels through the first 36 h after inclusion in the study were indicated in Table 2. for both intervention and control groups.

## Discussion

In the present randomized clinical trial, there were no statistically significant difference between the groups in terms of age, gender, heart rate and APACHE II score at the base line (Table 1.). 

Although, significant changes of IL-6 and CRP levels were observed following Acute Aortic Aneurysm (AAA) surgery, there were no significant changes in clinical endpoints such as heart rate, APACHE II scores, ICU length of stay and mortality rate.

Comparing intervention and control groups, IL-6 levels dramatically decreased after infusion of 10 g of MgSO4 over 12 h, represented by percentage change (%) from the baseline (-68.61±9.27 and -34.37 ± 8.78 respectively) which was statistically significant (P = 0.019). In addition, increasing of IL-6 within 12 h after the end of infusion in intervention group (+14.32%) in contrast with reduction in control group (-23.23%) indicates that the effect of MgSO4 diminishes rapidly following the end of infusion.

The effect of MgSO_4_ on IL-6 secretion has been formerly studied in the context of preeclampsia. Although MgSO_4_ has shown to decrease IL-6 secretion, the exact mechanism of action has not been established (11, 12). It is well known that Mg is a cation with numerous intracellular actions but some of its effects are mediated by extracellular mechanisms (13). IL-6 suppressing effect of Mg has not been clarified to be intracellular or extracellular. 

Based on our results, considering pharmacokinetic/pharmacodynamics relationship this effect is best to be explained by extracellular mechanisms. Following intravenous administration of large doses of MgSO_4_, Mg is primarily distributed in the vascular compartment and then it diffuses to extracellular fluid comportment. Due to slow equilibration of intracellular and extracellular Mg and short elimination half-life of serum ionized Mg, more than 95% of injected Mg is cleared through the renal system in first 24 h and very small quantities reach to intracellular compartment (14, 15). In this study, fast onset of action demonstrated by rapid decline of IL-6 following intravenous administration of Mg and short duration of action demonstrated by fast increment of IL-6 after the end of infusion suggests extracellular effects to be the most convenient mechanism of action. 

Previous studies have shown that IL-6 has an important role in the initiation of acute phase response by hepatocytes and consequently induces synthesis of CRP (5). Although it may be expected that lower amounts of IL-6 levels in intervention group would lead to decline in serum CRP, no correlation has been observed in our results. It may be due to short period of IL-6 being less in intervention group comparing with control group that might not be long enough to make a significant difference. Future studies with interventions which provide longer period of IL-6 suppression may display more clarified correlation between IL-6 and serum CRP level suppression. 

Considering the short duration of action of intravenous MgSO4 that has been observed in our study, the major question about its optimum dosing schedule seems to be “how long?” rather than “how much?”. Although there is no data about any benefit of continuing the infusion beyond 12 h to suppress IL-6 secretion, in the context of its extracellular mechanism of action, it would be reasonable to apply longer periods of continuous infusion to keep ionized serum Mg at higher levels for at least 24 h after AAA surgery until more conclusive data is available. In addition the rate of infusion may better to be based on our experiment (MgSO_4_ 20% 4 mL/h) until more extensive dose response studies are done; however, accurate monitoring of adverse effects such as hypotension, bradycardia and hypermagnesemia would be reasonable.

To our knowledge this was the first study of the evaluation of the effect of MgSO_4_ intravenous infusion with therapeutic doses on IL-6 and CRP levels in AAA patients. 

Limitations of this study could be mentioned as small sample size and limitations of serum ionized magnesium monitoring. 

Further studies could evaluate the optimum duration and rate of MgSO_4_ continuous infusion post AAA surgery and other conditions known to cause reperfusion injury. Another recommendation of future research is to evaluate pharmacokinetic/pharmacodynamics relationship of MgSO_4_ by means of repeated measurement of serum ionized Mg at baseline, during continuous infusion and early post infusion period.

## Conclusion

In this study continuous infusion of MgSO4 after AAA surgery showed anti-inflammatory effects mediated by IL-6 suppression. Short duration of action after the end of infusion suggests further benefit with application of longer periods of continuous infusion.

## References

[1] Sakalihasan N, Limet R, Defawe O (2005). Abdominal aortic aneurysm. Lancet.

[2] Aortic PDO, Tissue WC (2001). Pathogenesis of abdominal aortic aneurysms: a multidisciplinary research program supported by the National Heart, Lung, and Blood Institute. J. Vasc. Surg.

[3] Syk I, Brunkwall J, Ivancev K, Lindblad B, Montgomery A, Wellander E, Wisniewski J, Risberg B (1998). Postoperative fever, bowel ischaemia and cytokine response to abdominal aortic aneurysm repair—a comparison between endovascular and open surgery. Eur. J. Vasc. Endovasc. Surg.

[4] Dawson J, Cockerill GW, Choke E, Belli A-M, Loftus I, Thompson MM (2007). Aortic aneurysms secrete interleukin-6 into the circulation. J. Vasc. Surg.

[5] Salem M, Kasinski N, Munoz R, Chernow B (1995). Progressive magnesium deficiency increases mortality from endotoxin challenge: protective effects of acute magnesium replacement therapy. Crit. Care Med.

[6] Mirrahimi B, Hamishehkar H, Ahmadi A, Mirjalili MR, Aghamohamadi M, Najafi A, Abdollahi M, Mojtahedzahed M (2012). The efficacy of magnesium sulfate loading on microalbuminuria following SIRS: One step forward in dosing. DARU.

[7] Kruse H, Orent ER, McCollum E (1932). Studies on magnesium deficiency in animals I Symptomatology resulting from magnesium deprivation. ‎J. Biol. Chem.

[8] Harris TB, Ferrucci L, Tracy RP, Corti MC, Wacholder S, Ettinger Jr WH, Heimovitz H, Cohen HJ, Wallace R (1999). Associations of elevated interleukin-6 and C-reactive protein levels with mortality in the elderly. Am. J. Med.

[9] Ruilope LM, Redón J, Schmieder R (2007). Cardiovascular risk reduction by reversing endothelial dysfunction: ARBs, ACE inhibitors, or both? Expectations from the ONTARGET Trial Programme. Vasc. Health Risk Manag.

[10] Koenig W, Khuseyinova N, Baumert J, Thorand B, Loewel H, Chambless L, Meisinger C, Schneider A, Martin S, Kolb H, Herder C (2006). Increased concentrations of C-reactive protein and IL-6 but not IL-18 are independently associated with incident coronary events in middle-aged men and women: results from the MONICA/KORA Augsburg case-cohort study, 1984-2002. Arterioscler. Thromb Vasc. Biol.

[11] Amash A, Holcberg G, Sheiner E, Huleihel M (2010). Magnesium sulfate normalizes placental interleukin-6 secretion in preeclampsia. J. Interferon Cytokine Res.

[12] Xiao J, Yin Y, Shen F, Zhao J, Chen Q (2012). OS005 Treatment with magnesium sulphate reduced the serum level of IL-6 in preeclamptic women. Pregnancy Hypertens.

[13] Fomin VP, Gibbs SG, Vanam R, Morimiya A, Hurd WW (2006). Effect of magnesium sulfate on contractile force and intracellular calcium concentration in pregnant human myometrium. Am. J. Obstet. Gynecol.

[14] Cruikshank DP, Pitkin RM, Donnelly E, Reynolds WA (1981). Urinary magnesium, calcium, and phosphate excretion during magnesium sulfate infusion. Obstet. Gynecol.

[15] Jahnen-Dechent W, Ketteler M (2012). Magnesium basics. Clin. Kidney J.

